# Co-overexpression of two *Heat Shock Factors* results in enhanced seed longevity and in synergistic effects on seedling tolerance to severe dehydration and oxidative stress

**DOI:** 10.1186/1471-2229-14-56

**Published:** 2014-03-04

**Authors:** José-María Personat, Javier Tejedor-Cano, Pilar Prieto-Dapena, Concepción Almoguera, Juan Jordano

**Affiliations:** 1 Departamento de Biotecnología Vegetal, Instituto de Recursos Naturales y Agrobiología de Sevilla, Consejo Superior de Investigaciones Científicas (CSIC), 41012 Seville, Spain

**Keywords:** Combined overexpression, Drastic oxidative stress, Enhanced seed longevity, Heat Shock Factors, Severe dehydration, Stress tolerance, Transgenic tobacco

## Abstract

**Background:**

We have previously reported that the seed-specific overexpression of sunflower (*Helianthus annuus* L.) Heat Shock Factor A9 (*HaHSFA9*) enhanced seed longevity in transgenic tobacco (*Nicotiana tabacum* L.). In addition, the overexpression of *HaHSFA9* in vegetative organs conferred tolerance to drastic levels of dehydration and oxidative stress.

**Results:**

Here we found that the combined overexpression of sunflower Heat Shock Factor A4a (*HaHSFA4a*) and *HaHSFA9* enhanced all the previously reported phenotypes described for the overexpression of *HaHSFA9* alone. The improved phenotypes occurred in coincidence with only subtle changes in the accumulation of small Heat Shock Proteins (sHSP) that are encoded by genes activated by HaHSFA9. The single overexpression of *HaHSFA4a* in vegetative organs (which lack endogenous HSFA9 proteins) did not induce sHSP accumulation under control growth conditions; neither it conferred thermotolerance. The overexpression of *HaHSFA4a* alone also failed to induce tolerance to severe abiotic stress. Thus, a synergistic functional effect of both factors was evident in seedlings.

**Conclusions:**

Our study revealed that HaHSFA4a requires HaHSFA9 for *in planta* function. Our results strongly support the involvement of HaHSFA4a and HaHSFA9 in transcriptional co-activation of a genetic program of longevity and desiccation tolerance in sunflower seeds. These results would also have potential application for improving seed longevity and tolerance to severe stress in vegetative organs.

## Background

In the plant zygotic embryo, during orthodox seed maturation, different gene expression programs activate mechanisms that prevent and repair severe desiccation damage, at the same time allowing prolonged survival of the dry mature seed (reviewed [1-4] and references therein). Only the resurrection plants display similar levels of (dehydration and other abiotic) stress tolerance well beyond germination [5,6]. Interestingly, similar gene expression programs appear to be activated both in seeds and in vegetative organs of resurrection plants [7,8]. In sunflower, one of these genetic programs, which has been extensively studied in our lab, is under transcriptional control by Heat Shock Factors (HSFs); these HSFs include the seed-specific HaHSFA9 [9,10]. HaHSFA9 enhanced seed longevity in transgenic tobacco [11], when overexpressed from *DS10* sequences (a seed-specific promoter). We have also shown that the ectopic overexpression of *HaHSFA9* from *Cauliflower mosaic virus* (CaMV) *35S* sequences in tobacco seedlings conferred dramatic resistance of green organs and of whole seedlings to severe dehydration [12]. The tolerated dehydration was quantified as water loss of up to 98% of total water content. In addition, whole 35S:A9 seedlings resisted drastic oxidative stress conditions, as treatments in the dark with 200 mM H_2_O_2_ for 24 h [13]. The photosynthetic apparatus of the 35S:A9 seedlings, as well as other cellular membranes, resisted such stress conditions [13]. In all these instances, *HaHSFA9* overexpression activated genes that encode sHSPs from different classes. This resulted in the accumulation of cytosolic (CI, CII) and plastidial (P) sHSP forms. Most of the HaHSFA9-induced sHSPs are expressed mainly (or exclusively) during zygotic embryogenesis in seeds.

Precedent work in our lab indicated the existence of additional HSFs necessary for the activation of the HSFA9 program. Thus, stabilized forms of the auxin/indole acetic acid (Aux/IAA) protein HaIAA27 [14] or dominant-negative forms of HaHSFA9, but not inactive forms of HaHSFA9 [10], both caused reduction of seed longevity and loss of function of the HaHSFA9 program in tobacco seeds. We inferred that HaIAA27 would repress not only HaHSFA9, but also the additional HSFs that were first indicated by our results of loss of function using dominant-negative forms of HaHSFA9 [10]. The actual number of these additional HSFs is still unknown, but recently published results from our lab strongly indicated that HaHSFA4a is one of such HSFs [15]. HaHSFA4a showed *in planta* nuclear interaction with HaHSFA9; a synergistic transcriptional activation was observed on sunflower seed sHSP promoters, as *Hahsp17.6 G1*, when HaHSFA4a was assayed together with HaHSFA9; and, finally, the interaction of both HaHSFA9 and HaHSFA4a with HaIAA27 lead to passive repression of the synergism between HaHSFA9 and HaHSFA4a [14,15]. Based in these results, we have proposed that HaHSFA4a and HaHSFA9 might synergistically co-activate the same genetic program of seed longevity and desiccation tolerance in sunflower [15]. This program, referred to as the HSFA9 program, was functionally redundant with rest of programs that determine desiccation tolerance in seeds, programs that are inactive in vegetative organs [10].

Plant HSFs belong to different multigenic families (reviewed [16]). HSFs from these families, classes A, B, and C, differ among them and from other animal HSFs in short conserved sequences (signature sequences), and in structural features as the length and organization of the oligomerization domain (OD) and flexible linker sequences of variable length (15 to 80 amino acid residues) that connect the OD with the DNA-binding domain. The OD of class A HSFs has a characteristic insertion of 21 amino acid residues that extend the OD. This extended OD allows homo- and hetero-multimerization between class A HSFs [16]. The A4 HSFs (HSFA4) are characterized -among other properties- by the presence of conserved signature sequences (PVHSHS) located immediately after the DNA-binding domain (for example, [17]).

Overexpression of transcription factors has some advantages; thus, it is less affected by the functional redundancy that exists within multigenic families [18]. Furthermore, there are precedents where the co-overexpression of two transcription factors could reveal a synergistic enhancement of the phenotypes caused by one of the factors in separate (for example, [19,20]). Some plant *HSFs* have been characterized by overexpression. The reported *HSF* overexpression studies using transgenic plants mostly involve single, class A, *HSFs* from a brief list of species that, besides sunflower, it includes Arabidopsis, lily, rice, tomato and wheat (for example, [21-30]). We do not know of precedent studies that involve the conjoint overexpression of multiple *HSFs*.

Functional studies of plant HSFA4 are very scarce. There is only some evidence for HSFA4 functions related to moderate stress responses [31-33], as well as a single *HSFA4* overexpression study that we know of [26]. The later study has showed in transgenic rice plants that a *HSFA4* from rice (*OsHSFA4a*), or from wheat (*TaHSFA4a*), can confer Cd tolerance. Thus, the available studies for plant HSFA4 function have indicated their functional specialization.

In this work, we analyze the function of HaHSFA4a in transgenic tobacco. Tobacco is a plant closely related to sunflower, and we have showed that in tobacco transcriptional regulation of the HSFA9 program is conserved ([10,14], references therein). We overexpress *HaHSFA4a* alone, and in combination with *HaHSFA9*. We should emphasize that seeds or seedlings from the different non-transgenic (NT), single-transgenic, and double-transgenic lines, were subjected to the same, stress or seed deterioration conditions, in each case. We also point out that as in our previous studies [10-14,20], a molecular characterization of HSP accumulation was performed with seeds and seedlings grown under control (unstressed) conditions, the same for all lines compared. We thus tried further exploring the correlation of the observed stress protection with the HSPs that are present before the stress treatments. Seeds that combine the *DS10*-driven overexpression of *HaHSFA4a* and *HaHSFA9* resisted accelerated aging better than seeds that overexpress *HaHSFA9* only. The single, *DS10*-driven, overexpression of *HaHSFA4a* enhanced seed longevity. However, the *35S*-driven overexpression of *HaHSFA4a* alone did not induce any sort of abiotic stress tolerance in vegetative organs of seedlings. In contrast, the *35S*-driven overexpression of both *HaHSFA4a* and *HaHSFA9* caused further tolerance of seedlings to severe dehydration and to drastic oxidative stress conditions, as compared to the effect of HaHSFA9 alone. The enhanced stress tolerance occurred in coincidence with only subtle changes in the accumulation of small Heat Shock Proteins (sHSP). These results demonstrate *in planta* functional effects of HaHSFA4a on seed longevity and on tolerance to severe abiotic stress conditions. These effects, which are unmatched for a plant HSFA4, required HaHSFA9 (and/or seed-specific tobacco HSFs).

## Results

### Enhanced seed longevity in plants that conjointly overexpress HaHSFA9 and HaHSFA4a

**Figure 1:**
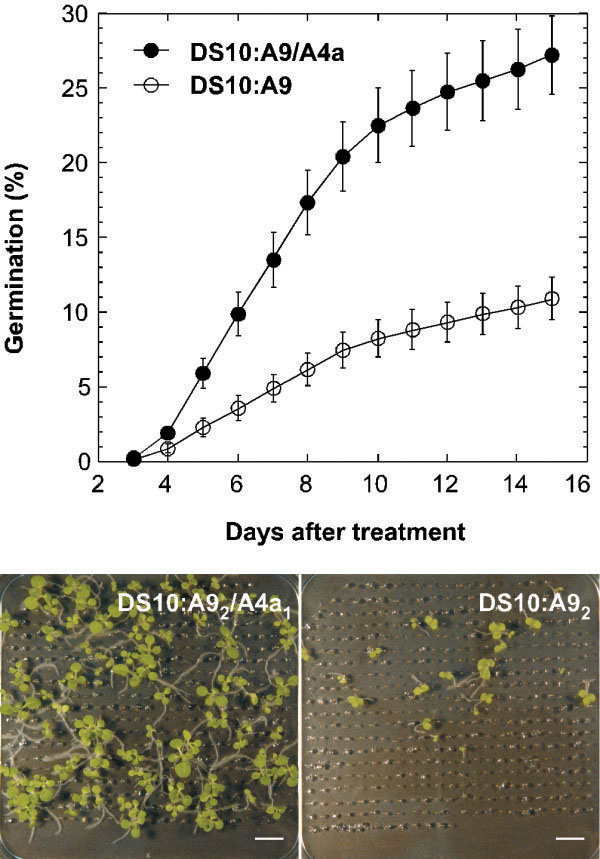
**The combined overexpression of *HaHSFA9 *and *HaHSFA4a *enhanced seed longevity beyond what achieved using only *HaHSFA9*.** Percent of germination (mean values ± SE) observed at different times after the aging treatments, at 52°C for 4 h, were compared between seeds of double homozygous DS10:A9/A4a and sibling, single homozygous DS10:A9 lines. The data correspond to three independent experiments performed with the seven pairs of sibling lines. Representative pictures of seedlings taken 15 days after the aging treatment are shown (bottom). Scale bars, 1 cm.

We have obtained lines that combine seed-specific overexpression of *HaHSFA9* and *HaHSFA4a*: the DS10:A9/A4a lines. We analyzed seven different sibling pairs of DS10:A9 (single-homozygous) and DS10:A9/A4a (double-homozygous) lines. We investigated whether the overexpression of *HaHSFA4a* in the DS10:A9/A4a lines enhances resistance to accelerated aging, a measure of seed longevity. We performed accelerated aging procedures similar to that used in our earlier studies, except that the aging temperature was increased from 50°C to 52°C. This was required to substantially age the DS10:A9 seeds, as with the 50°C treatments only sibling non-transgenic seeds were substantially affected [11]. The results of the experiments summarized in Figure 1, clearly show a statistically significant increase of the resistance to accelerated aging of the DS10:A9/A4a lines compared to the sibling DS10:A9 lines (F = 32.95, P < 0.0001, 1 and 831 df). We thus demonstrated in transgenic plants that the combined overexpression of *HaHSFA4a* and *HaHSFA9* enhanced seed-longevity beyond what observed for *HaHSFA9* in separate.

We also compared accelerated aging of single-homozygous DS10:A4a seeds with sibling NT seed. These aging treatments were also performed at 52°C, which allowed additional comparison with the experiments performed with the sibling DS10:A9 and DS10:A9/A4a lines (the results in Figure 1 explained above). The results of these experiments (Additional file 1) show that when only *HaHSFA4a* is overexpressed, this HSF enhances seed longevity. The comparison of data in Figure 1 with the results in Additional file 1 showed that seeds resisted the 52°C aging treatment in a similar way in the single-homozygous DS10:A4a lines as in the double-homozygous DS10:A9/A4a lines.

**Figure 2:**
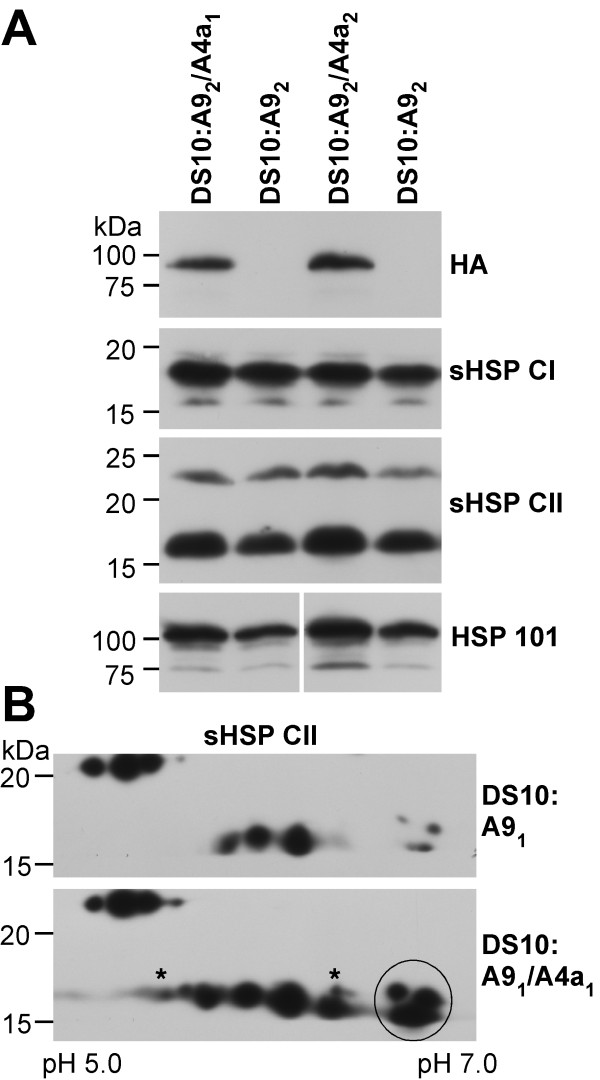
**Western analyses of HSP accumulation in seeds from double homozygous DS10:A9/A4a lines and sibling DS10:A9 lines.** The depicted line pairs represent the two A9 genetic backgrounds: A9_1_ and A9_2_. **(A)** 1D-western analyses; the antibodies used for immunodetection are indicated on the right. These include: HA (anti-hemaglutinin) and the anti-HSP antibodies specific for sHSP CI, sHSP CII and HSP101. **(B)** 2D-western analyses of sHSP CII accumulation. The asterisks and a thin circle mark polypeptides with enhanced accumulation in the DS10:A9_1_/A4a seeds. The pH range for isoelectric focusing is indicated (bottom). Molecular mass markers (in kDa) are indicated on the left.

In the DS10:A4a seeds, *HaHSFA4a* overexpression enhanced HSP accumulation. 1D-western blots showed clear effects on HSP101 and sHSP CII accumulation, and lesser effects on the sHSP CI. 2D-western-blots confirmed this and the specific augmented accumulation of some sHSP forms (Additional file 2). In the DS10:A9/A4a seeds a specific enhancement of HSP accumulation, respect to the sibling DS10:A9 lines, also occurred. In 1D-western blots, this enhancement was detected only for HSP101. The enhancement of specific sHSP-CII accumulation was observed only in 2D-western blots (Figure 2). We would like to point out that, as explained with detail in the Methods section, we performed careful controls to insure equal loading of total protein in all the 1D and 2D-western analyses included in this report.

### The single overexpression of HaHSFA4a does not induce stress tolerance in vegetative organs

**Figure 3:**
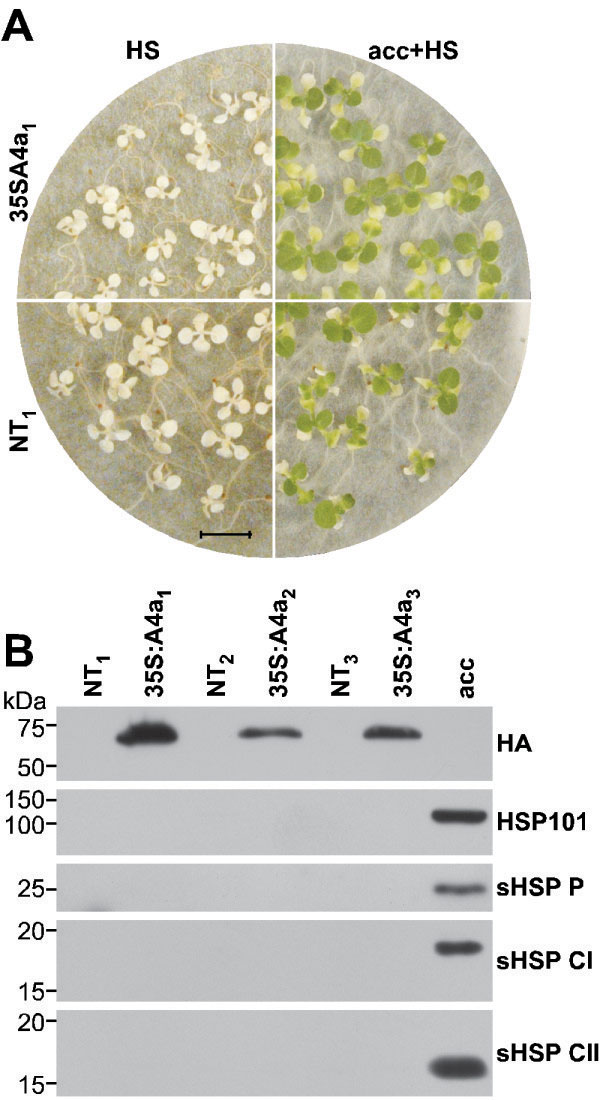
**Unaltered vegetative thermotolerance in the 35S:A4a seedlings. (A)** Representative results from three independent experiments (n = 39) performed with the three 35S:A4a sibling line pairs. (top left) The 35S:A4a_1_ seedlings did not survive direct exposure to 48°C for 2.5 h (HS). (bottom left) The sibling NT_1_ seedlings did not survive the same treatment. After an acclimation treatment for 3 h at 40°C (acc), both the 35S:A4a_1_ (top right) and the sibling NT_1_ seedlings (bottom right) acquire thermotolerance in a similar way and survive the 48°C treatment. Scale bar, 1 cm. **(B)** Western analyses of HSP and tagged-HaHSFA4a accumulation at normal growth temperatures in seedlings from the different line pairs. A sample from heat acclimated NT_3_ seedlings (acc) was used as positive control. Plastidial sHSP western detection (sHSP P). Rest of symbols as in Figure 2.

We analyzed the effect of the single overexpression of *HaHSFA4a*, on seedling stress tolerance, using the 35S:A4a lines. In these studies, three different, homozygous transgenic/non-transgenic (NT) sibling line pairs were used: 35S:A4a_1_, NT_1_; 35S:A4a_2_, NT_2_; and 35S:A4a_3_, NT_3_. We first analyzed tolerance to severe dehydration and to drastic oxidative stress conditions. The single overexpression of *HaHSFA4a*, in the 35S:A4a seedlings, failed to induce tolerance to the severe dehydration and the drastic oxidative stress conditions that withstand the 35S:A9 seedlings ([12,13], Additional file 3: A, B); protection of the photosystem II (PSII) was not observed in the 35S:A4a seedlings (Additional file 3: C). We also determined if the overexpressed *HaHSFA4a* affects the basal-, or the acquired-thermotolerance of the 35S:A4a seedlings. We used experimental conditions as previously reported for similar studies of the effects of HaHSFA9 [12]. Non-transgenic tobacco seedlings do not withstand lethal heat stress treatments for 2.5 h at 48°C. The 35S:A4a seedlings also did not resist the same 48°C treatment (Figure 3A). This result contrasts with what found for the 35S:A9 seedlings, where basal thermotolerance was enhanced and survival after a similar 48°C treatment was observed [12]. The NT seedlings acquired thermotolerance, and resisted the 48°C treatment, only after a heat-acclimation treatment for three hours at the non-lethal temperature of 40°C (Figure 3A). The 35S:A4a seedlings also acquired thermotolerance and subsequently resisted the 48°C treatment in a similar way as the sibling NT seedlings (compare the representative results in Figure 3A). HSP accumulation, including that of HSP101 and of different sHSPs (P, CI and CII) was not detected at normal growth temperatures in the 35S:A4a seedlings; these proteins however were detected at normal levels in the heat acclimated transgenic and sibling NT seedlings (Figure 3B; representative results shown for NT_3_).

### Enhanced tolerance to severe dehydration and to drastic oxidative stress in plants that conjointly overexpress HaHSFA9 and HaHSFA4a

**Figure 4:**
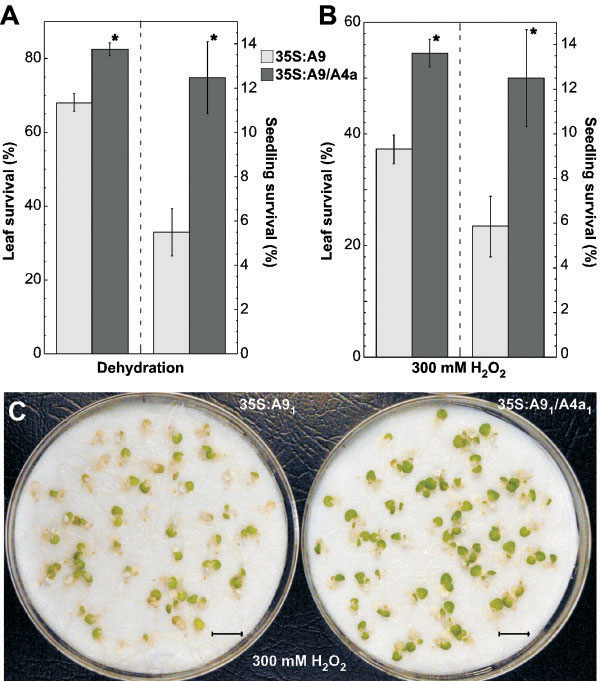
**Enhanced resistance to drastic dehydration and oxidative stress conditions in the 35S:A9/A4a seedlings.** Leaf-survival and whole seedling survival was evaluated. **(A)** Tolerance to severe dehydration. Data correspond to 17 independent experiments (n = 162) performed with the four sibling 35S:A9/A4a and 35S:A9 line pairs. **(B)** Tolerance to drastic oxidative stress conditions (treatments with 300 mM H_2_O_2_ for 24 h). Data correspond to 10 independent experiments (n = 100) performed with the same sibling line pairs. The dashed line in **(A)** and **(B)** separates the data that are described by the y-axis labels placed respectively to right or left in these panels. Data are mean values ± SE. Asterisks denote statistically significant differences (P ≤ 0.01). **(C)** Representative results shown for survival of leaves and whole seedlings after the H_2_O_2_ treatments. Scale bars, 1 cm.

We determined whether the combined overexpression of *HaHSFA9* and *HaHSFA4a* in transgenic tobacco enhances the stress tolerance observed upon the single overexpression of *HaHSFA9*[12,13]. The previously reported stress tolerance was unusually high; however there was room for further improvement. For example, the aerial part of transgenic tobacco seedlings survived dehydration better than roots [12]. This limited survival of whole seedlings after a water loss of ≈ 98% of the total initial water content. The analyses summarized in Figure 4 where performed with four sibling pairs of single-homozygous (35S:A9) and double-homozygous (35S:A9/A4a) lines. The combined overexpression of *HaHSFA9* and *HaHSFA4a* in the 35S:A9/A4a lines substantially enhanced survival of whole seedlings after either stress treatment: severe dehydration (Figure 4A), or treatments with 300 mM H_2_O_2_ (Figure 4B). In both cases, survival of whole-35S:A9/A4a seedlings more than doubled that of 35S:A9 siblings. These differences were statistically highly significant (Figure 4A, t = -3.59, P = 0.0004; Figure 4B, t = -2.59, P = 0.01; see “seedling survival”). The, surviving, whole-35S:A9/A4a seedlings represented slightly above 12% of the initial amount of seedlings. However, in most seedlings only some leaves resisted the stress treatments. Survival after dehydration of one to four leaves per seedling (Figure 4A) was also significantly higher for the 35S:A9/A4a lines compared to the 35S:A9 lines (t = -4.82, P < 0.0001). After the 300 mM H_2_O_2_ stress treatments, only up to two true leaves per seedling survived, such survival (Figure 4B) was also higher for the 35S:A9/A4a lines compared to the 35S:A9 lines (t = -4.87, P < 0.0001). Figure 4C shows pictures with a representative example of the results summarized in Figure 4B.

**Figure 5:**
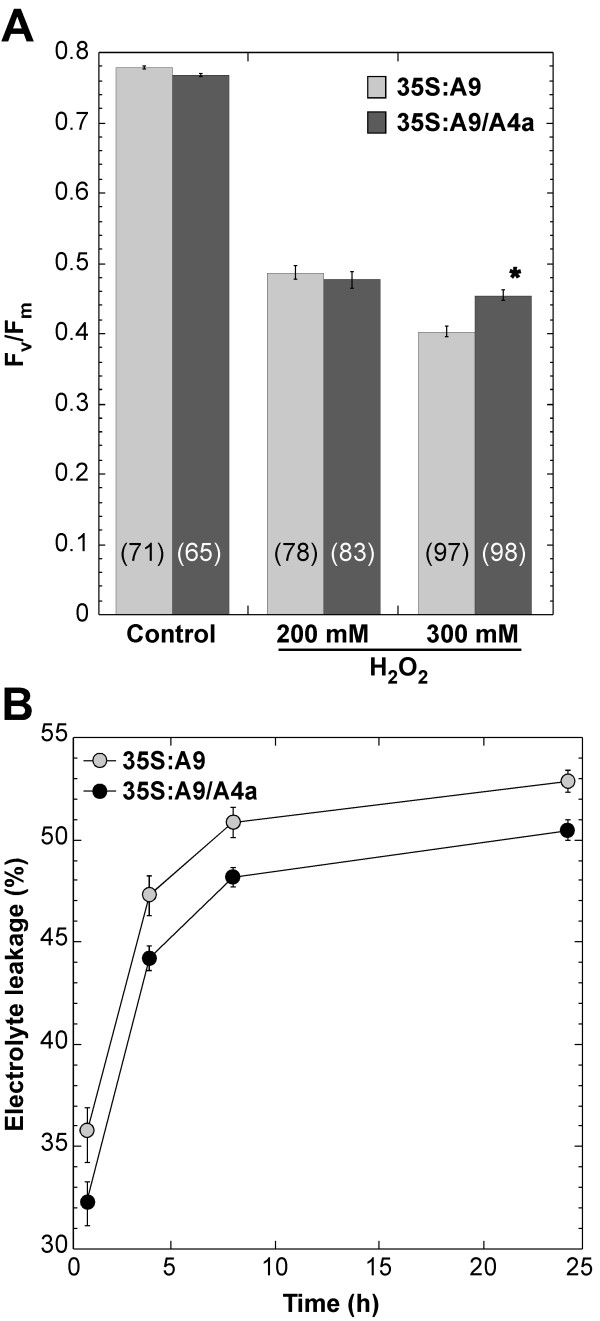
**Enhanced protection of the PSII and of cellular membranes in the 35S:A9/A4a seedlings.** Results from experiments performed with the same sibling line pairs as in Figure 4. Data are mean values ± SE. **(A)** Comparison of maximum quantum yield (F_v_/F_m_) of PSII. Sample sizes are indicated with bracketed numbers within the shaded bars. Asterisks denote statistically significant differences (P ≤ 0.01), observed only after the 300 mM H_2_O_2_ treatment. **(B)** Diminished electrolyte leakage (EL) in the 35S:A9/A4a seedlings. Results from two independent experiments (n = 17). EL was determined in deionized (MilliQ) water at different times between 1 h and 24 h.

Enhanced protection of the PSII, as evaluated with F_v_/F_m_ values in the 35S:A9/A4a lines compared with sibling 35S:A9 lines, was also observed (Figure 5A), but only after the 300 mM H_2_O_2_ stress treatments (F = 23.21, P = 0.0001). After, standard, 200 mM H_2_O_2_ treatments [13], there was not difference between the F_v_/F_m_ of these lines (F = 0.236, P = 0.63). The additional protection of the PSII conferred by the combination of HaHSFA9 and HaHSFA4a is thus observed only under very drastic, oxidative stress, conditions. The 35S:A9/A4a seedlings also showed lower electrolyte leakage under normal grown conditions, when compared to 35S:A9 siblings (Figure 5B). This supports the enhancement of protection of other cellular membranes (photosynthetic and non-photosynthetic) in the 35S:A9/A4a seedlings. We conclude that the overexpression of *HaHSFA4a*, in combination with that *HaHSFA9*, further enhanced the, already unusually high, stress resistance conferred by the single overexpression of *HaHSFA9*. Furthermore, the functional effects of HaHSFA4a in vegetative organs required HaHSFA9. We could show that the tagged HaHSFA4a protein was detected even at slightly higher level in the 35S:A4a than in the 35S:A9/A4a seedlings (Additional file 4). Thus, a functional interaction-specificity for HSFs as HaHSFA9, rather than the expression level of *HaHSFA4a* would explain the lack of effects of HaHSFA4a when singly overexpressed in seedlings.

**Figure 6:**
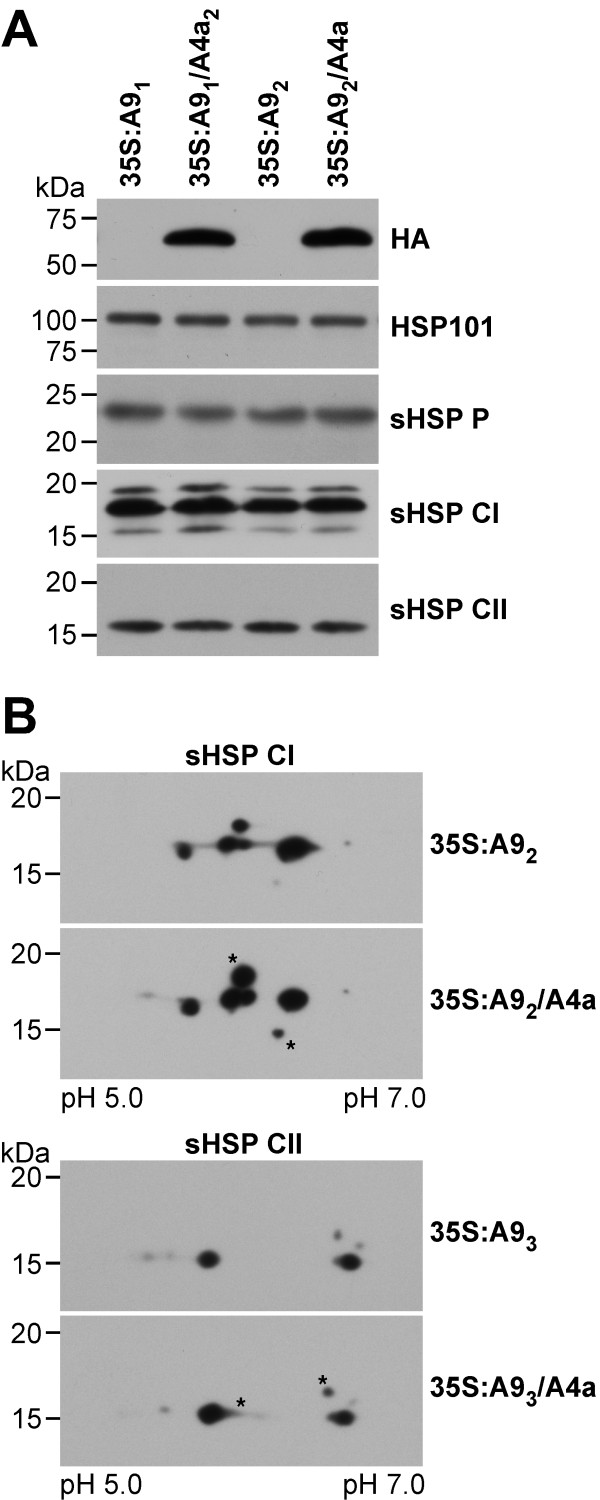
**HSP accumulation in 35S:A9/A4a seedlings compared to 35S:A9 siblings. (A)** 1D-western analyses with sample from the indicated sibling line pairs. The antibodies used for immunodetection are indicated on the right. **(B)** Representative 2D-western analyses of sHSP CI (top) and sHSP CII (bottom) accumulation. The asterisks mark polypeptides with enhanced accumulation in the 35S:A9/A4a seedlings. Rest of symbols as in Figure 2.

The combined overexpression of *HaHSFA9* and *HaHSFA4a* in the 35S:A9/A4a seedlings, resulted only in a slight enhancement of specific HSP-accumulation at normal growth temperatures. This was observed upon very careful comparison with sibling 35S:A9 material. Among the analyzed HSPs (HSP101, sHSP-P, sHSP-CI and sHSP-CII) only some cytosolic sHSPs (CI and CII) were affected; furthermore, this slight accumulation enhancement was detected using 2D gels, but not with 1D-gels (Figure 6). We think that it is unlikely that the observed enhancement of vegetative stress tolerance was caused by these sHSPs; these results would rather point to alternative or complementary effects of, still unknown, (i.e., non-*HSP*) genes coactivated by HaHSFA9 and HaHSFA4a.

## Discussion

To date, synergistic interactions of plant HSFs that enhance transcriptional activation have been analyzed only by transient expression [15,34,35]. We note that, except for our precedent study [15], these interactions involve “vegetative HSFs” (constitutive or heat-induced). The interactions between “vegetative HSFs” would be thus relevant mostly for heat-, and other moderate stress responses in organs other than seeds. As far as we know, our work represents the first report addressing the effects of combined overexpression of *HSFs* in transgenic plants. We could thus show that, in vegetative organs of seedlings, HaHSFA9 and HaHSFA4a showed synergistic functional effects on tolerance to severe dehydration and to drastic oxidative stress (Figures 4 and 5). These results would functionally confirm that at least two HSFs, HaHSFA9 and HaHSFA4a, co-activate the same program of seed longevity and desiccation tolerance in sunflower.

There is a single report [26] that describes the effect of overexpression of *HSFA4* from wheat (*TaHSFA4a*) and rice (*OsHSFA4a*, *OsHSFA4d*). The overexpression of *TaHSFA4a* in rice plants conferred Cd tolerance. Unpublished observations cited in the same report suggest that TaHSFA4a is not involved in thermotolerance [26]. In addition, TaHSFA4a and OsHSFA4a, but not a similar monocot HSFA4, OsHSFA4d, conferred Cd tolerance in yeast [26]. Loss of function analyses of Arabidopsis *AtHSFA4c* and rice *OsHSFA4d* has indicated additional HSFA4 functions. These functions include the sensing of moderate oxidative stress and gravitropism, thus being also unrelated to the conventional heat stress response and to thermotolerance [31-33]. The work reported here for *HaHSFA4a* includes the first described effects of overexpression of a dicot *HSFA4*. HaHSFA4a, as TaHSFA4a [26], does not seem to be involved in canonical heat responses or in thermotolerance. However, the functional effects of HaHSFA4a seem to be quite different from what was known for similar HSFA4. HaHSFA4a would be specifically involved in seed functions related to longevity and in tolerance to severe dehydration.

The observed functional effects of HaHSFA4a appear to require at least HaHSFA9 and/or other seed-specific HSFs that are not present in vegetative organs, either in unstressed or heat-stressed conditions. This functional requirement would set apart HaHSFA4a from the rest of plant, class A, activator HSFs analyzed to date. The overexpression of *HaHSFA4a* in vegetative organs of seedlings potentiated phenotypes that we previously described for the overexpression of *HaHSFA9,* but only when HaHSFA9 was conjointly overexpressed. *HaHSFA4a* overexpression also enhanced seed longevity, which is a *HaHSFA9* overexpression phenotype that we also have confirmed by loss of function [10]. These results agree with a hypermorphic effect of HaHSFA4a on HaHSFA9, which would safely support the suggested novel functions for HaHSFA4a. Because of the normal phenotype of the 35S:A4a plants, the potentiated phenotypes of the 35S:A9/A4a plants are explained as a synergistic enhancement of the effects of HaHSFA9. In tobacco seeds, where an endogenous *HSFA9* is expressed [10], the single overexpression of *HaHSFA4a* enhanced seed-longevity and HSP accumulation (Additional files 1 and 2). *HaHSFA4a* also augmented seed longevity when overexpressed together with *HaHSFA9* (Figure 1). However, this effect was similar to what observed in the single transgenic DS10:A4a lines (Additional file 1), and thus appears to be largely dependent on endogenous HSFs (including HSFA9) and not of the overexpressed *HaHSFA9*. The levels of the endogenous HSFA9 protein would be high and thus sufficient to account for the observed HaHSFA4a effect. Indeed, and consistently with this interpretation, the HaHSFA9 protein appears to be quite abundant in sunflower seed embryos [9]. In contrast, in seedlings in absence of the endogenous HSFA9 protein, the single overexpression of *HaHSFA4a* did not enhance thermotolerance; neither it induced accumulation, at normal growth temperatures, of HSPs as sHSP-CI, -CII, -P, and HSP101 (Figure 3). These results indicate that HaHSFA4a failed to functionally interact with the tobacco HSFs that are involved in vegetative thermotolerance; this would include the constitutive HSFs present in seedlings, and the HSFs induced by the heat-acclimation treatment used in Figure 3. This inference from the results in Figure 3 would agree with the specificity that HaHSFA4a showed in its synergistic interaction with HaHSFA9, but not with LpHSFA2, in transient assays [15]. The lack of effect of HaHSFA4a, on HSP accumulation and thermotolerance, contrasts with what observed upon the single overexpression of other class A *HSFs*; this includes, for example, *HSFA1b *(formerly named *HSF3*), *HSFA2*, *HSFA3*, and *HSFA9* of Arabidopsis and other plants [12,21-25,27,28,30]. In seedlings, the enhancement of dehydration and oxidative stress tolerance by HaHSFA4a was strictly dependent on the conjoint overexpression of *HaHSFA9* (compare Figure 3 and Additional file 3). Our results agree with reported transient expression analyses using *sHSP-CI* promoters in sunflower. These analyses showed that HaHSFA4a had very little (if any at all) transcriptional activity by itself [15]. In contrast, HaHSFA4a assayed together with HaHSFA9 showed a strong synergistic transcriptional effect; furthermore, HaHSFA4a and HaHSFA9 physically interact with each other [15]. Therefore, these two HSFs might cause in transgenic plants the observed functional effects as hetero-oligomers. Our results do not exclude the involvement in the same genetic program of additional *HSFs* besides *HSFA9* and *HSFA4a* in tobacco, sunflower and related plants. However, if additional *HSFs* able to functionally interact with HaHSFA4a exist in tobacco, these *HSFs* would be, as *HSFA9*, preferentially (or exclusively) expressed in seeds.

## Conclusions

Our work demonstrated a novel involvement of HaHSFA4a in seed longevity and severe stress tolerance, as well as the strict dependency on HaHSFA9 (or similar tobacco HSF) of the functional effects of HaHSFA4a. These findings contribute to the very scarce previous knowledge on plant type A4 HSF (HSFA4) function. The single overexpression of *HaHSFA4a* did not alter vegetative stress tolerance in transgenic tobacco. In contrast, HaHSFA4a enhanced seed longevity. Furthermore, in vegetative organs of seedlings HaHSFA9 and HaHSFA4a showed synergistic functional effects on tolerance to severe dehydration and to drastic oxidative stress. We thus showed potentially useful effects when HaHSFA4a and HaHSFA9 (or similar tobacco HSF) are combined. Our results might open new ways to engineering seed longevity and tolerance of plants to severe dehydration and to drastic oxidative stress conditions.

## Methods

### Generation of the, transgenic, DS10 lines

A 3xHA-tagged form of *HaHSFA4a* was integrated in a binary plasmid derived from pSK-ds10EC1 [36] and pBIB-Hyg [37]. This binary plasmid was named pBIB-*DS10:3xHA:HaHSFA4a:DS10*. The *HaHSFA4a* cDNA was amplified by PCR from pBI221:*HaHSFA4a*[15]. In this step, an *Xba*I site (located 3 bp before the ATG) and a *Sal*I site (located 5 bp after the STOP codon) were introduced with the oligonucleotides 5′-GTTGTTGGTATATCTAGATCAATGATGAATGATGTTCATGG-3′ and 5′-GTAAATTTAGACAGTCGACCATTATCAACTTCTCTCTACTG-3′ (with an annealing temperature of 67°C). The amplified DNA (1215 bp) was digested with *Xba*I and *Sal*I. The resulting fragment (1178 bp) was introduced between the *Xba*I and *Sal*I sites of the pUC19-35S:HA vector [10], thus generating the pUC-35S:*3xHA:HaHSFA4a* plasmid. The *3xHA:HaHSFA4a* cassette was amplified by PCR from this plasmid with the oligonucleotides 5′-TCTAGTAAAAATGGCATACC-3′ and 5′-TTATCAACTTCTCTCTACTG-3′. The amplified 1339 bp fragment was introduced in the Klenow-filled *EcoR*I site of pSK-ds10EC1 [36], thus originating the pSK-ds10EC1:*3xHA:HaHSFA4a* plasmid. This plasmid was digested with *Sal*I and *Xba*I, and the resulting 4967 bp fragment was cloned between the corresponding sites of pBIB-Hyg [37], which originated, pBIB-*DS10:3xHA:HaHSFA4a:DS10*, the tagged DS10:A4a binary plasmid.

To obtain the, single-transgenic, DS10:A4a lines, we transformed tobacco with the tagged DS10:A4a binary plasmid. Using procedures that we have described in detail for DS10:A9 lines [11], except that selection of transgenic plants was on media with 50 μg mL^-1^ hygromycin B, we obtained four different pairs of DS10:A4a lines (homozygous, single-transgenic) and sibling non-transgenic (NT) lines: DS10:A4a_1_, NT_1_; DS10:A4a_2_, NT_2_; DS10:A4a_3_, NT_3_; and DS10:A4a_4,_ NT_4_.

To obtain the, double-transgenic, DS10:A9/A4a lines, two homozygous, DS10:A9, transgenic lines that overexpress *HaHSFA9* from *DS10* (a seed-specific promoter) were transformed with the tagged DS10:A4a binary plasmid. In this case, the parental, DS10:A9, transgenic lines were previously described as DS10:A9#6-7 and DS10:A9#14-5 [11]. We first obtained heterozygous DS10:A4a lines (with single integration events) in the two homozygous DS10:A9 backgrounds. The double-homozygous DS10:A9/A4a lines were obtained by segregation on media with 50 μg mL^-1^ hygromycin B in the subsequent generation. We also selected for the sibling DS10:A9 lines, which were used as the proper, single-transgenic control, lines. The selection procedures were described in detail for similar DS10 line pairs [20]. This resulted in the following seven, sibling, line pairs: DS10:A9_1_/A4a_1_, DS10:A9_1_; DS10:A9_1_/A4a_2_, DS10:A9_1_; DS10:A9_2_/A4a_1_, DS10:A9_2_; DS10:A9_2_/A4a_2_, DS10:A9_2_; DS10:A9_2_/A4a_3_, DS10:A9_2_; DS10:A9_2_/A4a_4_, DS10:A9_2_; and DS10:A9_2_/A4a_5_, DS10:A9_2_. The A9_1_ and A9_2_ backgrounds correspond to DS10:A9#6-7 and DS10:A9#14-5, respectively. In the A9_1_ background, we obtained two, different, double-homozygous lines; five double-lines were obtained in the A9_2_ background.

### Generation of the, transgenic, 35S lines

A 2284 pb, Hind*III*-Kpn*I,* DNA fragment excised from the pUC-35S-3xHA:*HaHSFA4a* plasmid (see above) was cloned between the Hind*III* and Kpn*I* sites of pBIB-Hyg [37]. As a result, we obtained pBIB-Hyg-35S-3xHA:*HaHSFA4a*, the tagged 35S:A4a binary plasmid. We transformed tobacco (var. Xanthi) with the tagged 35S:A4a binary plasmid. Using the procedures described for the selection of 35S:A9 line pairs [12], except that selection of transgenic plants was on media with 50 μg mL^-1^ hygromycin B, we obtained three different pairs of 35S:A4a lines (homozygous, single-transgenic) and sibling NT lines: 35S:A4a_1_, NT_1_; 35S:A4a_2_, NT_2_, and 35S:A4a_3_, NT_3_.

Three homozygous transgenic lines that overexpress *HaHSFA9* from CaMV 35S sequences were transformed with the tagged 35S:A4a binary plasmid. The parental transgenic lines were previously described as 35S:A9#2-18, 35S:A9#12-4, and 35S:A9#17-8 [12]. We first obtained heterozygous 35S:A4a lines (with single integration events) in the three homozygous 35S:A9 backgrounds. The double-homozygous 35S:A9/A4a lines were obtained by segregation in the subsequent generation. We also selected for the, respective, sibling 35S:A9 lines, which were used as the proper, single-transgenic control, lines. We thus obtained four pairs of sibling lines: 35S:A9_1_/A4a_1_, 35S:A9_1_; 35S:A9_1_/A4a_2_, 35S:A9_1_; 35S:A9_2_/A4a, 35S:A9_2_, and 35S:A9_3_/A4a, 35S:A9_3_. The A9_1_, A9_2_ and A9_3_ backgrounds correspond to 35S:A9#2-18, 35S:A9#12-4, and 35S:A9#17-8 respectively. In the A9_1_ background, we obtained two different double-homozygous lines, 35S:A9_1_/A4a_1_ and 35S:A9_1_/A4a_2._

### Seed longevity and seedling stress tolerance assays

Seed sterilization, germination, and seedling growth under controlled conditions were as described [11]. Germination of seeds after accelerated aging treatments was performed as previously reported [11], except that the treatments were for 4 h at 52°C.

Stress tolerance was analyzed in 3–4 week-old seedlings grown on Petri dishes with MS media. We performed severe dehydration (DT2) and oxidative stress treatments with H_2_O_2_ in the dark for 24 h, using conditions essentially as described [12,13], respectively]. The H_2_O_2_ concentration was increased from 200 mM to 300 mM, to decrease survival of the 35S:A9 seedlings after the oxidative stress treatments. Thermotolerance (tolerance to high temperature) was analyzed using procedures that have been described for similar analyses of the 35S:A9 lines [12].

### Chlorophyll fluorescence

The maximal quantum efficiency (F_v_/F_m_) of PSII was determined as the ratio of variable fluorescence (F_v_) to maximum fluorescence of dark-adapted state (F_m_). Chlorophyll fluorescence was measured with a mini-PAM Photosynthesis Yield Analyzer (Heinz Walz, Effeltrich, Germany); procedures were essentially as previously described [13].

### Electrolyte leakage

Electrolyte leakage (EL) was measured using an EC-Meter GLP 31+ conductivimeter (CRISON). Seedlings from the same Petri dish (50–60 seedlings) were placed in 25 mL of Milli-Q water and incubated with gentle shaking at room temperature for different times. Cumulative EL for each sample and time point was determined. Finally, the samples were autoclaved and the water brought back to room temperature to determine the total (100%) leakage values.

### Analyses of HSP accumulation

Western blots, after 1D- or 2D-electrophoresis, were performed using the procedures [11] and the HSP-specific antibodies that we previously described. We carefully adjusted the samples used in the 1D and 2D-western analyses for equal total protein amounts in the different comparisons. For 1D-westerns total protein content of samples was first estimated by Bradford assays and then verified by Ponceau S staining of the proteins transferred to the PVDF membranes. Examples of these loading controls are shown in Additional file 5. For 2D-westerns we used samples that were first quantified by Bradford and 1D gel assays, as for the 1D-westerns. The representative 2D-western results shown in Figures 2 and 5 and Additional file 2 were selected to show the protein spots that consistently increased in intensity respect other non-variable spots. We previously demonstrated the specificity of the anti-sHSP CI and anti-sHSP CII antibodies generated in our lab. These antibodies showed class-sHSP specificity: the anti-sHSP CI antibodies do not recognize sHSP CII proteins and vice versa [38]. The commercial anti-HSP21 antibody (Agrisera, AS08-285) detects only sHSP P proteins, but not sHSP CI and sHSP CII proteins [13]. The anti-HSP101 antibody (Agrisera, AS07-253) is an anti-HSP101/ClpB N-terminal antibody that only detects heat-induced HSP101 proteins, but not the constitutive HSP101 proteins [20]. The anti-HA-peroxidase antibodies (high affinity 3F10) do not detect native plant proteins, under either control or stress conditions [20].

### Statistical analyses

In experiments were data showed normal distribution or could be normalized by logarithmic transformation (Figures 1 and 5, and Additional files 1 and 3), we used ANOVA. The ANOVA (Figure 5A and Additional file 3) and repeated-measures ANOVA analyses (Figures 1 and 5B, and Additional file 1) were as described with detail in a former publication from our lab [11]. Alternatively, t-Student tests were used when data could not be normalized (Figure 4), similarly to what we have previously reported (see [14], Table S1). F and t are the statistics respectively associated to the ANOVA and t-Student tests. In Figures 1, 4 and 5, we averaged the data for the different pairs of analogous sibling lines. In these cases, the statistical analysis of differences between the averaged data was consistent with the results obtained when the differences were separately analyzed for each sibling line pair.

## Abbreviations

Aux/IAA: Auxin/indole acetic acid protein; CaMV: Cauliflower mosaic virus; EL: Electrolyte leakage; HSFs: Heat Shock Factors; HaHSFA4a: (*Helianthus annuus* L.) Heat Shock Factor A4a; HaHSFA9: (*Helianthus annuus* L.) Heat Shock Factor A9; HSP: Heat Shock Protein(s); OD: Oligomerization domain; PSII: Photosystem II; sHSP: Small Heat Shock Protein(s).

## Competing interests

The authors declare have no competing interests.

## Authors’ contributions

JMP performed most of the experimental work with the help of PPD and CA. JTC contributed with the initial transformation and characterization of the different transgenic lines. PPD performed the statistical analyses. JJ conceived and coordinated the study and wrote the manuscript. PPD and CA edited the manuscript. All authors read and approved the final manuscript.

## Supplementary Material

Additional file 1 **The single overexpression of *HaHSFA4a *in tobacco seeds enhanced seed longevity.** Percent of germination observed at different times after the aging treatments; comparison between seeds of homozygous DS10:A4a and sibling, non-transgenic (NT) lines. Click here for file

Additional file 2 **Western analyses of HSP accumulation in seeds from different DS10:A4a line pairs. (A)** 1D-western analyses using the following antibodies: anti-hemaglutinin and the anti-HSP antibodies specific for sHSP CI, sHSP CII and HSP101. **(B)** 2D-western analyses of sHSP CI accumulation. **(C)** 2D-western analyses of sHSP CII accumulation. Click here for file

Additional file 3 **The 35S:A4a seedlings did not resist drastic dehydration and oxidative stress conditions.** Percent of seedlings with one or more surviving leaf and whole seedling survival after the stress treatments. Data are mean values ± SE. **(A)** Tolerance to severe dehydration. **(B)** Tolerance to drastic oxidative stress conditions. **(C)** Comparison of maximum quantum yield [Fv/Fm] of PSII after treatments with H_2_O or with 200 mM H_2_O_2_ for 24 h. Click here for file

Additional file 4 **Comparison of the accumulation levels of the tagged HaHSFA4a protein in the 35S:A4a and 355:A9/A4a seedlings.** 1D-western analyses using anti-hemaglutinin antibodies. Click here for file

Additional file 5 **Examples of total protein loading controls for the protein samples analyzed by western blot in this article.** Ponceau S stained PVDF membranes. Click here for file
