# Supplementary material for: Co-overexpression of two Heat Shock Factors results in enhanced seed longevity and in synergistic effects on seedling tolerance to severe dehydration and oxidative stress
Source: BMC Plant Biol. 2014 Mar 4;14:56. doi: 10.1186/1471-2229-14-56 (PMC4081658; doi:10.1186/1471-2229-14-56)
Supplement: Additional file 1 — The single overexpression of HaHSFA4a in tobacco seeds enhanced seed longevity. Percent of germination observed at different times after the aging treatments; comparison between seeds of homozygous DS10:A4a and sibling, non-transgenic (NT) lines. [file 1471-2229-14-56-S1.pdf]

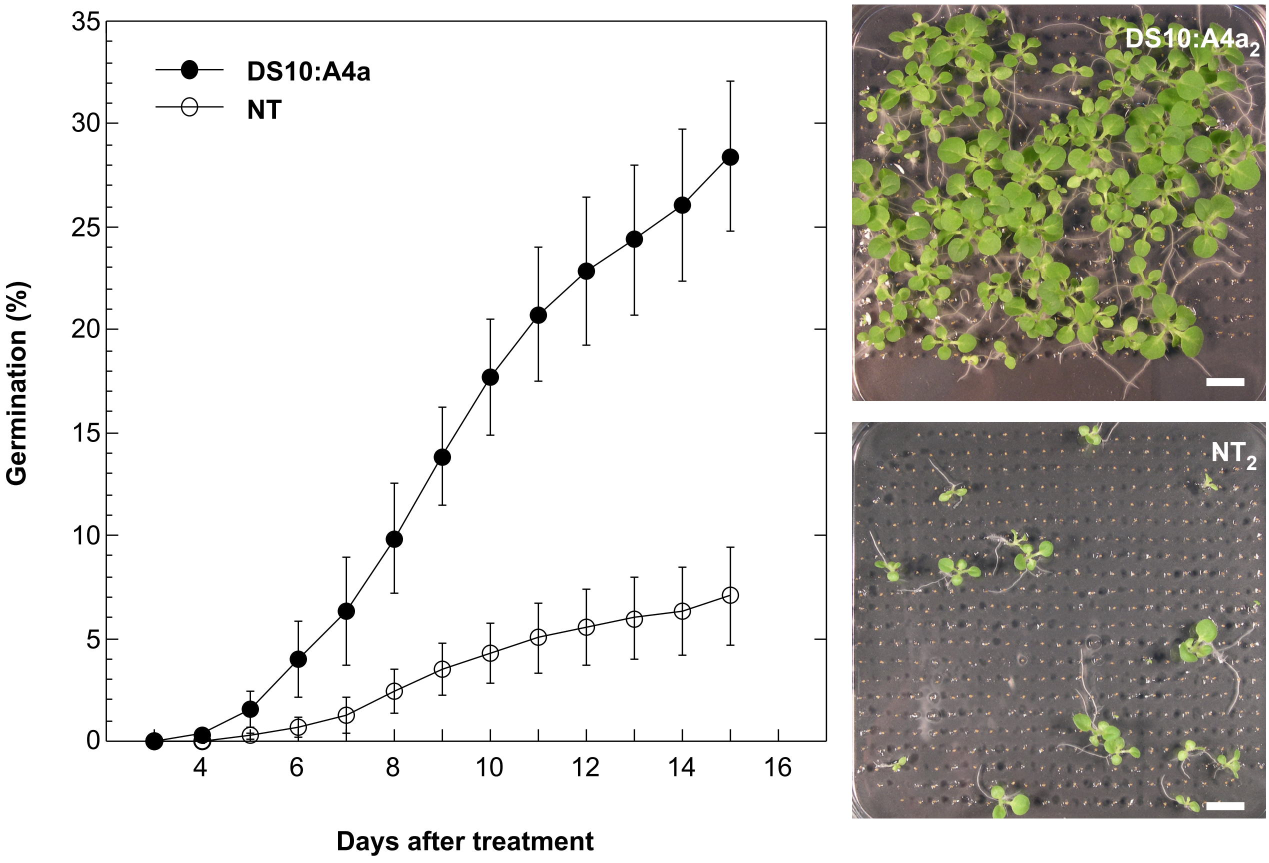

Additional file 1: The single overexpression of *HaHSFA4a* in tobacco seeds enhanced seed longevity. Percent of germination observed at different times after the aging treatments, at 52 ºC for 4 h, were compared between seeds of homozygous DS10:A4a and sibling, non-transgenic (NT) lines. The data (mean values ± SE) correspond to two independent experiments performed with the four pairs of sibling lines. Representative pictures of seedlings taken 15 days after the aging treatment are shown on the right. Scale bars, 1 cm.
