# Supplementary material for: Co-overexpression of two Heat Shock Factors results in enhanced seed longevity and in synergistic effects on seedling tolerance to severe dehydration and oxidative stress
Source: BMC Plant Biol. 2014 Mar 4;14:56. doi: 10.1186/1471-2229-14-56 (PMC4081658; doi:10.1186/1471-2229-14-56)
Supplement: Additional file 2 — Western analyses of HSP accumulation in seeds from different DS10:A4a line pairs. (A) 1D-western analyses using the following antibodies: anti-hemaglutinin and the anti-HSP antibodies specific for sHSP CI, sHSP CII and HSP101. (B) 2D-western analyses of sHSP CI accumulation. (C) 2D-western analyses of sHSP CII accumulation. [file 1471-2229-14-56-S2.pdf]

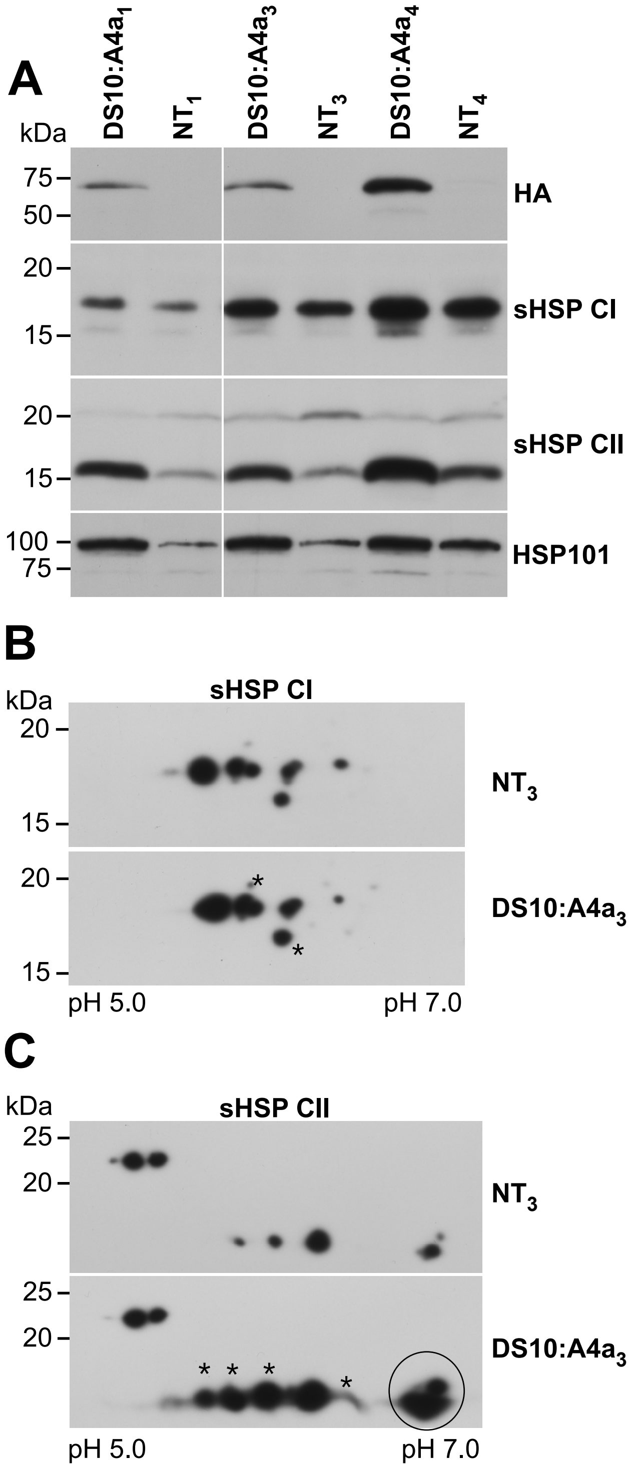

Additional file 2: Western analyses of HSP accumulation in seeds from different DS10:A4a lines and the corresponding non-transgenic sibling lines -NT-. (A) 1D-western analyses; the antibodies used for immunodetection are indicated on the right. These include: HA (anti-hemaglutinin) and the anti-HSP antibodies specific for sHSP CI, sHSP CII and HSP101. **(B)** 2D-western analyses of sHSP CI accumulation. (**C)** 2D-western analyses of sHSP CII accumulation. The asterisks and a thin circle mark polypeptides with enhanced accumulation in the DS10:A4a seeds. The pH range for isoelectric focusing in 2D-gels is indicated. Molecular mass markers (in kDa) are indicated on the left.
