# Supplementary material for: Co-overexpression of two Heat Shock Factors results in enhanced seed longevity and in synergistic effects on seedling tolerance to severe dehydration and oxidative stress
Source: BMC Plant Biol. 2014 Mar 4;14:56. doi: 10.1186/1471-2229-14-56 (PMC4081658; doi:10.1186/1471-2229-14-56)
Supplement: Additional file 3 — The 35S:A4a seedlings did not resist drastic dehydration and oxidative stress conditions. Percent of seedlings with one or more surviving leaf and whole seedling survival after the stress treatments. Data are mean values ± SE. (A) Tolerance to severe dehydration. (B) Tolerance to drastic oxidative stress conditions. (C) Comparison of maximum quantum yield [Fv/Fm] of PSII after treatments with H2O or with 200 mM H2O2 for 24 h. [file 1471-2229-14-56-S3.pdf]

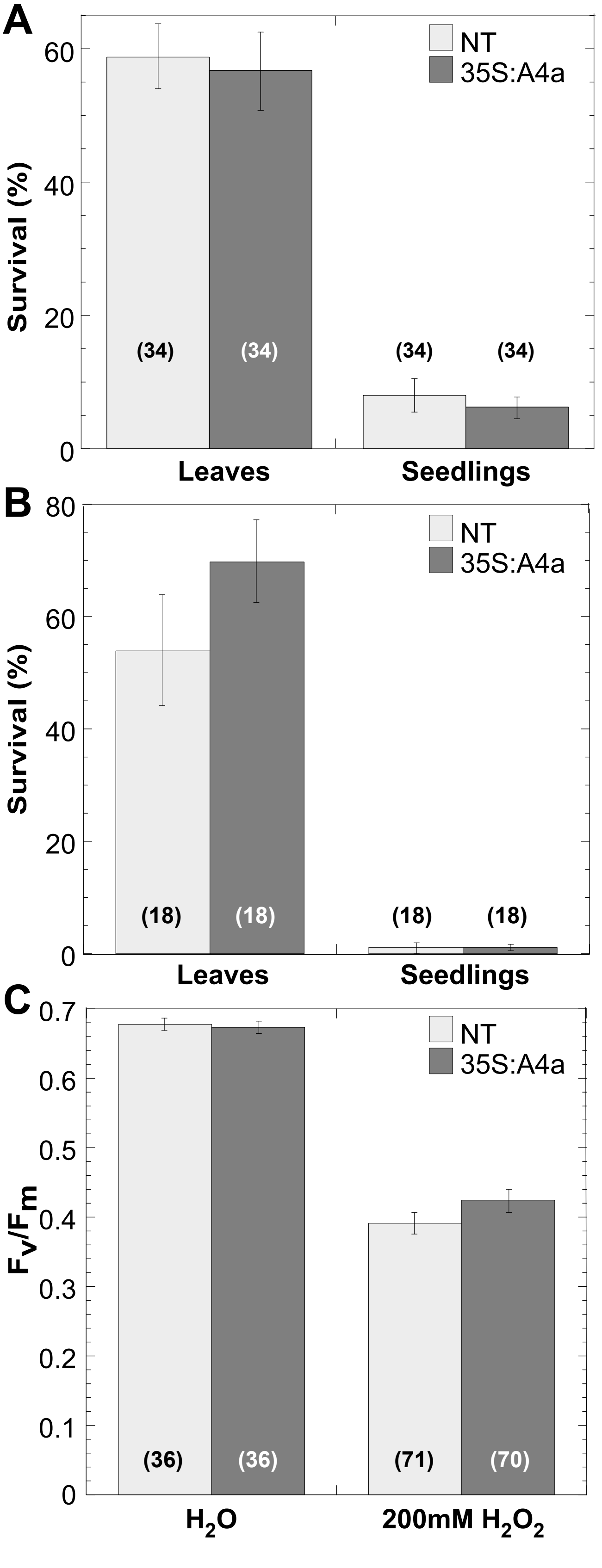

Additional file 3: The 35S:A4a seedlings did not resist drastic dehydration and oxidative stress conditions. We evaluated survival of leaves (the % of seedlings with one or more surviving leaf) and whole seedling survival after the stress treatments. We represent data from three independent experiments performed with the three 35S:A4a sibling line pairs. Sample sizes are indicated with bracketed numbers in the bars within each panel. **(A)** Tolerance to severe dehydration evaluated with DT2 treatments. (**B)** Tolerance to drastic oxidative stress conditions after treatments with 200 mM H_2O_2 for 24 h. (**C)** Comparison of maximum quantum yield [Fv/Fm] of photosystem II after treatments with H_2O or with 200 mM H_2O_2 for 24 h. Data are mean values ± SE. No statistically significant differences were observed.
