# Supplementary material for: Co-overexpression of two Heat Shock Factors results in enhanced seed longevity and in synergistic effects on seedling tolerance to severe dehydration and oxidative stress
Source: BMC Plant Biol. 2014 Mar 4;14:56. doi: 10.1186/1471-2229-14-56 (PMC4081658; doi:10.1186/1471-2229-14-56)
Supplement: Additional file 4 — Comparison of the accumulation levels of the tagged HaHSFA4a protein in the 35S:A4a and 355:A9/A4a seedlings. 1D-western analyses using anti-hemaglutinin antibodies. [file 1471-2229-14-56-S4.pdf]

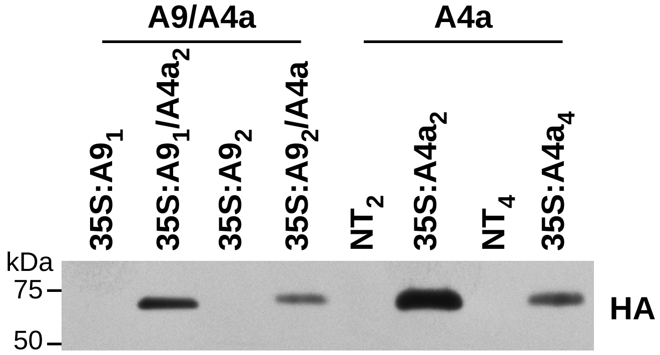

Additional file 4: Comparison of the accumulation levels of the, HA-tagged, HaHSFA4a protein in the 35S:A4a and 355:A9/A4a seedlings. 1D-western analyses of 25 μg total protein from the indicated lines. Detection of HaHSFA4a using anti-hemaglutinin antibodies (HA). Molecular mass markers (in kDa) are indicated on the left.
