# Supplementary material for: Co-overexpression of two Heat Shock Factors results in enhanced seed longevity and in synergistic effects on seedling tolerance to severe dehydration and oxidative stress
Source: BMC Plant Biol. 2014 Mar 4;14:56. doi: 10.1186/1471-2229-14-56 (PMC4081658; doi:10.1186/1471-2229-14-56)
Supplement: Additional file 5 — Examples of total protein loading controls for the protein samples analyzed by western blot in this article. Ponceau S stained PVDF membranes. [file 1471-2229-14-56-S5.pdf]

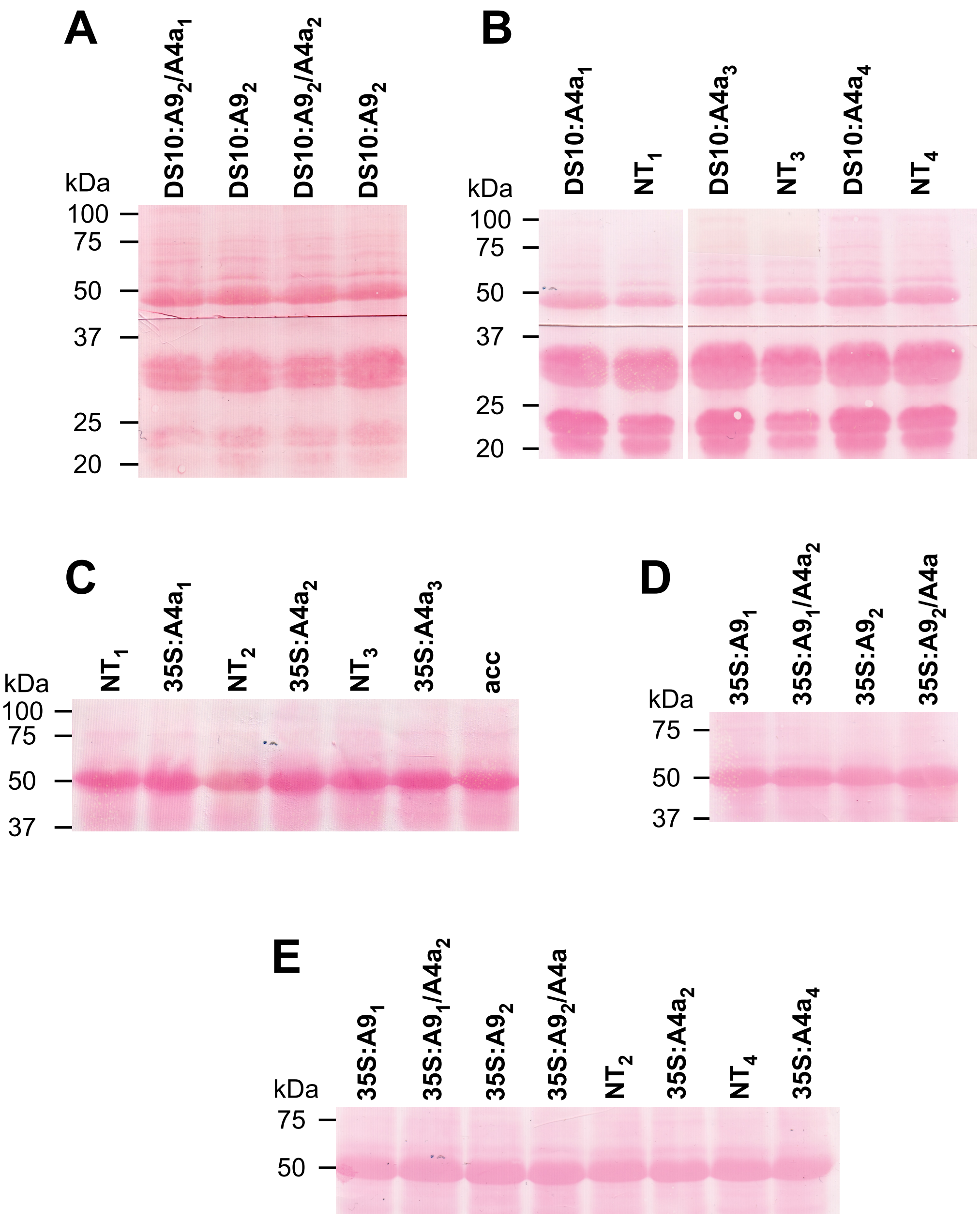

Additional file 5: Examples of total protein loading controls for the protein samples analyzed by western blot in this article. Ponceau S stained PVDF membranes corresponding to the total protein samples analyzed in Figure 2 **(A)**, Additional file 2 **(B)**, Figure 3B **(C)**, Figure 6A **(D),** and Additional file 4 **(E)**. Molecular mass markers (in kDa) are indicated on the left. The RbcL protein (≈ 50 kDa) was the sole distinct band stained in the seedling protein samples **(C, D, E)**. Other abundant proteins were stained in the seed samples **(A, B)**.
